# Use of non-invasive neurostimulation for rehabilitation in functional movement disorders

**DOI:** 10.3389/fresc.2022.1031272

**Published:** 2022-11-16

**Authors:** Talyta Grippe, Naaz Desai, Tarun Arora, Robert Chen

**Affiliations:** ^1^ Edmond J. Safra Program in Parkinson’s Disease, Morton and Gloria Shulman Movement Disorders Clinic, Toronto Western Hospital, UHN, Toronto, ON, Canada; ^2^ Division of Neurology, University of Toronto, Toronto, ON, Canada; ^3^ Neuroscience Graduate Program, Federal University of Minas Gerais, Belo Horizonte, Brazil; ^4^ Krembil Research Institute, University Health Network, Toronto, ON, Canada

**Keywords:** neuromodulation, functional movement disorder, rehabilitation, transcranial magnetic stimulation, transcranial direct current stimulation, transcutaneous electrical stimulation, low intensity focus ultrasound

## Abstract

Functional movement disorders (FMD) are a subtype of functional neurological disorders which involve abnormal movements and include multiple phenomenologies. There is a growing interest in the mechanism, diagnosis, and treatment of these disorders. Most of the current therapeutic approaches rely on psychotherapy and physiotherapy conducted by a multidisciplinary team. Although this approach has shown good results in some cases, FMD cause a great burden on the health system and other treatment strategies are urgently needed. In this review, we summarize past studies that have applied non-invasive neurostimulation techniques, such as transcranial magnetic stimulation (TMS), transcranial direct current stimulation (tDCS) and peripheral nerve stimulation as a treatment for FMD. There is an increasing number of studies related to TMS including randomized controlled trials; however, the protocols amongst studies are not standardized. There is only preliminary evidence for the efficacy of non-invasive neuromodulation in reducing FMD symptoms, and further studies are needed. There is insufficient evidence to allow implementation of these techniques in clinical practice.

## Introduction

Functional neurological disorder is a complex neuropsychiatric syndrome with neurological symptoms that cannot be explained by a lesion or related to an identified dysfunction of the central nervous system (1). It has been suggested that it is the result of underlying neuropsychiatric disturbances that drive the disorder (2). In functional neurological disorder, the primary pathophysiological processes are considered to be alterations in the functioning of brain networks rather than abnormalities of brain structures (3). Functional movement disorders (FMD) are a subtype of functional neurological disorders which involve abnormal movements and include multiple phenomenologies. Tremor is the most frequent abnormal movement, but FMD can also manifest as dystonia, myoclonus, gait disorders, parkinsonism, tics, stereotypies, facial movements, chorea, and may include more than one type of abnormal movements (4, 5). FMD represents a major public health and economic problem, with an estimated global annual incidence of 4–12 cases per 100,000 (6).

The diagnosis of FMD has improved with the use of more objective clinical signs, the concept of positive diagnosis rather than a rule-out approach, and with the help of ancillary techniques such as electrophysiology (7). However, the diagnosis of FMD remains challenging, frequently leading to misdiagnosis and delay in treatment.

The treatment of FMD presents an even greater challenge than the diagnosis. Therapeutic options can be broadly classified into pharmacologic and non-pharmacologic. Pharmacologic options mainly involve the use of antidepressants and the therapeutic benefits of these remain limited (8, 9). The more established non-pharmacological options for treatment of FMD include psychotherapy (mainly cognitive behavioral therapy) alone or in combination with physical (PT), occupational (OT), or speech therapy (8, 10–12). A group of FMD experts recommended that PT treatment be based on a biopsychosocial etiological framework in which heterogeneous mixtures of predisposing, precipitating and perpetuating factors are considered and formulated, with the acceptance that relevant factors differ between different patients (11). There is growing evidence of the benefits of interdisciplinary approach in FMD treatment (13).

Other forms of non-pharmacological interventions that have been used include non-invasive neuromodulation techniques, such as transcranial magnetic stimulation (TMS), transcranial direct current stimulation (tDCS), transcutaneous electrical nerve stimulation (TENS), and functional electrical stimulation (FES) (8, 14–16). These techniques have shown promising therapeutic effects in neurorehabilitation and neuropsychiatric disorders, supporting their potential use as treatment of FMD (17, 18), which are considered as networks disorders. Non-invasive neurostimulation techniques may target specific nodes that are involved in FMD including the motor cortex, prefrontal cortex, supplementary motor area, temporoparietal junction, insula and amygdala (19).

Here, we review the central and peripheral neurostimulation protocols that have been used in the treatment of FMD and evaluate their efficacy, limitations, and possible future clinical applications.

## Non-invasive brain stimulation

### Transcranial magnetic stimulation

TMS is a non-invasive technique that induces electrical currents in the targeted brain regions through magnetic pulses that passes the scalp and skull virtually unattenuated. Repetitive TMS (rTMS) refers to the application of trains of TMS pulses that can induce neuroplasticity leading to neuromodulatory effects (20). High frequency (5 Hz or more) rTMS or intermittent theta burst stimulation (iTBS) increases cortical excitability. In contrast, low-frequency rTMS (∼1 Hz) or continuous theta burst stimulation (cTBS) decreases cortical excitability (20, 21). The stimulation intensity is often adjusted relative to the motor threshold (MT), which refers to the minimum intensity that consistently elicits motor-evoked potentials in the target muscle. MT measured at rest is referred to as the resting motor threshold (RMT). The stimulation intensity can be subthreshold (<100% MT) or suprathreshold (>100% MT). The United States Food and Drug Administration (FDA) has approved different TMS protocols for the treatment of major depressive disorder, migraine and obsessive-compulsive disorder (22).

In major depressive disorder, the rTMS protocol with level A evidence (definite efficacy) is high-frequency rTMS on the left dorsolateral prefrontal cortex (DLPFC), which was tested in two multicentered, randomized clinical trials (22). The standard treatment parameters are frequency of 10 Hz, using a figure-of-8 coil and 75 trains of pulses with 4 s duration and 15–26 s of intertrain interval, at intensity of 120% RMT (23). Moreover, the use of iTBS to the left DLPFC is considered equivalent to high intensity rTMS from a non-inferiority study and both protocols are approved by the FDA (24). There is level B evidence (probable efficacy) for low-frequency rTMS (1 Hz) to the right DLPFC using a figure-of-8 coil, one single train of pulses with 20 min duration and intensity of 120% RMT (23, 25). This approach was tested in more than one placebo-controlled study and was considered equivalent to high-frequency rTMS in a non-inferiority trial (25, 26).The treatment duration is usually at least 2 weeks, and the reduction of depressive symptoms for 4–6 weeks (25).

There are increasingly more number of studies that are evaluating the use of rTMS as treatment for movement disorders, including Parkinson's disease, dystonia, and tremor (27). Below we discuss literature related to the use of rTMS in FMD.

An open-label study evaluated 24 consecutive inpatients which included patients with functional dystonia, myoclonus, tremor, Parkinsonism, and stereotypies. The rTMS protocol had an average of 20 stimuli (120% RMT) delivered at a low frequency of 0.25 Hz over the motor cortex either contralateral to the symptomatic side or bilaterally if symptoms were bilateral. The results showed that 75% of patients had more than 50% improvement in the FMD score, defined as a combination of the Abnormal Involuntary Movement Scale and the walking sub-score of the disability score from the Burke–Fahn–Marsden dystonia rating scale immediately after the intervention. Ten patients (42%) relapsed during the follow-up period but after every new rTMS session, they all improved to a similar extent compared to after the first session. At the last follow-up with a median time of 19.8 months, 71% of the patients reported improvement as indicated by a score lower than 4 in the Global Clinical Impression (GCI) Scale. The GCI scale ranges from 1 (very much improved) to 7 (very much worse) (28). A subsequent randomized-controlled trial by the same group compared motor cortex rTMS with a control condition of spinal roots magnetic stimulation (RMS). Repetitive RMS (rRMS) was performed over the cervical (upper limbs) or lumbar (lower limbs) spinal roots ipsilateral to the FMD affected body region. rRMS was chosen as the control treatment because it mimicked rTMS-induced movement but did not directly stimulate the cortex. rTMS was delivered on the lateral (upper limbs) or medial (lower limbs) motor cortex contralateral to the FMD-affected body region. This study used an average of 50 consecutive stimuli at 120%–150% of the RMT for rRMS. The rTMS was delivered at a low frequency (0.25 Hz) in 33 patients. The treatments were delivered in a cross-over design. Seventeen participants received rTMS on day one and rRMS on day 2, and 16 participants received interventions in the opposite order. They were evaluated immediately after each stimulation and at day 3 to observe the combined effects of rTMS and rRMS. There was no significant difference in the degree of improvement between the two groups after the first session of rTMS (median improvement: 37.5%) or rRMS (median improvement: 23.6%). On day 3, 22 of the 33 patients showed greater than 50% improvement on the primary outcome measure (FMD score); however, there was no significant difference in the final percentage improvement between the two groups (29). At 1-year follow-up, 56% of 29 patients evaluated reported improvement measured by GCI of 2 or lower. Relapse of symptoms occurred in 12 patients with a median time of 6 months after the first intervention. The patients who relapsed were offered another rTMS session at least 3 months after the previous session, and all these patients improved significantly after another session of rTMS. The authors concluded that improvement in FMD was likely due to a cognitive-behavioral effect rather than cortical neuromodulation (29).

Another open-label study evaluated the effects of the suggestion of benefit combined with motor cortex and premotor cortex rTMS in six patients with chronic (>2 years) FMD, which included patients with tremor, dystonia, myoclonus, and gait disorder. rTMS was initially performed for five consecutive days at 0.33 Hz (total of 50 pulses per day) at 90% RMT over the dominant motor cortical representation of the first dorsal interosseus (FDI) muscle in all patients regardless of the FMD presentation. After two weeks, the participants were assessed on quality-of-life measures including the four domain scores of the World Health Organization Quality of Life brief scale (WHOLQOL-BREF)—physical health, psychological, social relationships, and environment, and on CGI rated by the patient and by the investigators. One month later, a similar protocol was applied with rTMS targeted to the premotor cortex (PMC) and the patients were reassessed two weeks later. Suggestion of the benefit of rTMS was introduced at the initial baseline visit and was reinforced at each subsequent study visit. The suggestion was given using a standardized presentation and patients were told that they had a very high likelihood of benefiting from rTMS. The results showed improvement in the physical domain of the WHOLQOL-BREF scale after both protocols compared to baseline but a worsening in the psychological domain following the PMC protocol. Only one subject showed substantial improvement on self-rated CGI after PMC stimulation, whereas no significant change on this scale was noted in any of the patients when evaluated by the investigators (30).

Another randomized, controlled trial in 18 individuals with functional tremor involved five consecutive days of 1 Hz rTMS at intensity of 90% RMT (1,600 biphasic pulses per day). Nine patients received rTMS targeted to the motor cortex contralateral to the affected limb (region representing the FDI muscle for upper limbs and tibialis anterior muscle for lower limbs) or bilaterally if the tremor was bilateral. For the control intervention, the other 9 patients received intervention through a sham coil that provided an acoustic stimulus comparable to that of the real coil was used. After the first intervention, the “mean” Psychogenic Movement Disorders Rating Scale (PMDRS) score and the Tremor subscores of this scale decreased in both groups, but changes from baseline were significant only in the active rTMS group after 2 months (31). A second open-label phase included 3 weekly hypnosis sessions, each lasting about 1 h and combined with a rTMS session. Significant changes from baseline values were maintained in phase 2 after 12 months for the active rTMS group. However, for the control group, neither the mean PMDRS total nor the Tremor subscores differed significantly from baseline after 6 or 12 months (31). As secondary outcomes, the CGI showed perception of improvement in active group but no significant change in the control group, the Hospital Anxiety and Depression Scale (HAD) and the Hamilton Depression Rating Scale (HDRS) mean scores remained unchanged in both groups throughout the 1-year follow-up (31). An open-labeled study also evaluated patients with functional tremor using a protocol of 30 rTMS pulses over the hand area of the primary motor cortex contralateral to the affected hand(s) at a frequency of 0.2 Hz. The first half of the pulses were administered at the intensity of 120% RMT, and the second half at 140% RMT. rTMS treatment led to a significant reduction in tremor frequency measured by kinematic sensors and video evaluations of spontaneous tremor and tremor while performing a distraction task (tapping at a frequency different from the tremor frequency with the contralateral arm). The duration of symptom relief was transient in seven participants, whereas four participants had lasting symptom relief for up to 12 months (32).

A proof-of-concept open-labeled study evaluated the effects of iTBS targeting the left dorsolateral prefrontal cortex in six patients with FMD. The patients received three iTBS sessions, and each session consisted of 600 pulses in 50 Hz bursts of three pulses, separated by 200 ms (5 Hz frequency) for 2 s, followed by 8 s of no stimulation over 190 s. There was a 20-minute interval between each session. The stimulation intensity was 120% RMT. The abnormal movements in the patients included tremor, myoclonus, abnormal gait, and dystonia. The subjects were assessed with functional magnetic resonance imaging (fMRI) while presented with stimuli with different valences from the Karolinska Directed Emotional Faces database and a modified version of the Simplified-Functional Movement Disorders Rating Scale (S-FMDRS). There was a significant decrease in the fronto-amygdala connectivity and a change in amygdala reactivity to emotionally valenced stimuli assessed with fMRI. Moreover, a mixed-linear model showed significant decrease in S-FMDRS score from pre to post-iTBS and at the final evaluation performed 24 h after the last session compared to the pre-iTBS scores at the first session (33).

There are many case reports on the use of rTMS for the treatment of FMD. One case with functional blepharospasm and another case with functional hemiparesis were treated with 12 single-pulses of TMS targeting the vertex at 30%–80% of stimulator output. Both participants experienced complete recovery, with recurrence of mild symptoms at three months and six months, respectively (34). Another report of a patient with functional dystonia associated with dissociative post-traumatic stress disorder used a combined treatment of 1 Hz rTMS of the cingulate cortex as the main target, and supplementary motor area and PMC as secondary targets. After 36 sessions, the patient showed a reduction in depression, anxiety and dystonia measured by the Beck Depression Inventory 2 (BDI-2), Generalized Anxiety Disorder 7 (GAD-7), Patient Health Questionnaire 9 (PHQ-9), Beck Anxiety Inventory (BAI), Toronto Western Spasmodic Torticollis Rating Scale (TWSTRS), and a perception of improvement evaluated by the CGI (35). In another case report, a patient with functional myoclonus in the right lower limb was treated with daily sessions of 1 Hz rTMS with 1,200 biphasic magnetic pulses over the left PMC five times a week for 6 weeks, with a significant reduction in frequency and intensity of the involuntary movements for up to two months as measured by the Unified Myoclonus Rating Scale (UMRS) (36). One patient with an unusual presentation of functional tetraparesis, mixed tremors and functional seizures was treated with biofeedback psychotherapy together with rTMS (20 sessions, two sessions per day for 10 days; 1 Hz, 150% RMT, 20-minute sessions, 300 pulses per session). Each session was targeted one of the four limbs. The coil was positioned on the right/left side to target the upper (lateral motor cortex) or lower (medial motor cortex) limbs. The patient visualized motor activities of her limbs during stimulation of the motor cortex. Symptoms improved between the 4th and 6th day of treatment with increase in muscle power, followed by further improvement of other functional symptoms. At 2-years post-treatment, the patient had no functional neurological symptoms (37).

The majority of the rTMS studies for FMD used the motor cortex or PMC as the main target showing promising results with reduction of the severity of functional motor symptoms. These results represent some preliminary evidence for the use of rTMS as a therapeutic approach for FMD. However, it is important to acknowledge that most of the studies were open-labelled and only few had a sham-controlled design. For the studies with a sham-controlled condition, the sham condition only allowed for masking of sound and did not include masking for scalp sensation. In summary, large randomized controlled studies with well-designed sham conditions are needed to assess the efficacy of rTMS as a treatment for FMD.

### Transcranial direct current stimulation

Transcranial direct-current stimulation (tDCS) is a non-invasive brain stimulation (NIBS) technique that involves the use of weak electric stimulation (1–2 mA for 5-to-30-minutes) for modulation of neural activities. The increase or decrease in neuronal excitability causes alterations in cerebral functions that can be exploited for therapeutic purposes (38). In a simplified model, positive stimulation (anodal tDCS) results in excitatory effect whereas negative stimulation (cathodal tDCS) leads to hyperpolarization of cell membrane with inhibitory effect on the brain excitability. A 10-minute session generates effects that last about one hour (39). An evidence-based guideline for tDCS concluded that there was no indication with level A evidence (definitive efficacy), but level B evidence (probable efficacy) was granted for the treatment of several neurological and psychiatric disorders, including fibromyalgia, depression, and craving/addiction (40).

The rationale for the use of tDCS in the treatment of depressive disorders is similar to the use of TMS and is related to the proposed imbalance of excitability between left and right dorsolateral and ventromedial prefrontal cortex (41). The current approach is to enhance neural activity in the left DLPFC with anodal stimulation and/or to reduce neural activity in the right DLPFC with cathodal stimulation. Recent trials supported the use of 30 min of tDCS delivered at 2 mA with the cathode placed over the right DLPFC, with reduction in depressive symptoms in 45% of the treated population for up to 3 months (42, 43).

Regarding the use of tDSC in FMD patients, an exploratory study evaluated the effects of a single 20-minute session of anodal tDCS at 1.5 mA intensity compared to sham stimulation (stimulation for 30 s to reproduce the itching or tingling sensation at the beginning with the stimulator turned off for the next 19 min) over the right posterior parietal cortex. The study was performed in nine participants with functional motor symptoms and seven healthy controls. Each group received both interventions in a randomized order with at least 2 days between them. The outcomes were interoceptive sensitivity and the ability to accurately perceive visceral afferent information measured as heartbeat perception. A similar degree of improvement in interoceptive sensitivity was observed after both real and sham tDCS (44). Another study evaluated the effects of two sessions of anodal tDCS of the right temporoparietal junction together with yoga compared to sham stimulation (18 s of stimulation and then turned off) in 5 patients with FMD, who presented with tremor, myoclonus, dystonia, and gait disorders. The participants received both sham and active tDCS in a randomized order, with a washout period of 3 weeks. The results showed no difference in the CGI, modified Rankin scale (mRS) and Stigma Scale for Chronic Illness (SSCI-8) scores between the two groups (45).

These tDCS studies for FMD did not show significant results when comparing the active with the sham protocols. The short duration of the intervention, the heterogeneity of the outcome measures, and the presence of other interventions such as yoga in association with the tDCS are relevant points to consider when interpreting the results of tDCS studies. Moreover, tDCS may be coupled with another intervention such as physical therapy when treating conditions like motor deficits after stroke (40). This approach could be further explored for treatment of FMD with tDCS.

## Non-invasive peripheral electrical stimulation

A variety of peripheral electrical stimulation have been used as treatment for FMD in the late 20th and early 21st century, and have frequently reported to be successful (18). There is some evidence for the use of transcutaneous electrical nerve stimulation (TENS) and functional electrical stimulation (FES) for movement disorders.

TENS devices deliver low-voltage electrical currents to the skin. Although the mechanisms of action of TENS and the optimal parameters have not been established, some data showed that TENS transiently affects motor and sensory thresholds, suggesting that it modulates both motor and sensory transmission within the central nervous system (46). Pain is the most commonly studied indication for TENS. A recent meta-analysis which included several randomized controlled trials (RCT) concluded that there was moderate-certainty evidence for this therapeutic tool to reduce pain. The stimulation parameters used were variable and included frequency up to 250 pulses per second, pulse duration up to 500 µs and amplitude up to 60 mA (47). Previous studies have shown a potential effect of TENS in the treatment of movement disorders, including dystonia, tremor and FMD (48–50).

An open-label study examined the use of TENS as a treatment in 15 participants with FMD, which included patients with functional tremor, dystonia, myoclonus, gait disturbance or speech disorder. The TENS therapy used self-adhesive electrodes placed 5 cm apart over the muscles that were most affected by FMD. TENS was delivered in 2 s trains, separated by 2 s pauses, administered at a frequency of 150 Hz during a total of 30 min daily. The patients were evaluated after the first session and up to 4 months later. Five out of 15 participants demonstrated a robust (>50%) improvement in PMDRS score after the first session of TENS, and these patients had no FMD symptoms at the follow-up evaluation. Two patients had a transient worsening in the PMDRS scores (<25%). The mean PMDRS score of all the patients evaluated at follow-up was significantly reduced compared to baseline (from 35.3 to 11.6). The patient-rated PMD severity regarding magnitude (from 8.7 to 5.2), persistence (from 8.8 to 5.7) and associated disability (from 7.2 to 4.8) also showed significant reduction (51).

One case reported functional improvements with the use of TENS (80 Hz, 150 µs pulse width) in a patient with medication refractory propriospinal myoclonus of probable functional origin. The electrodes were placed to deliver electrical current over the area innervated by the low thoracic spinal segments. The stimulation intensity was gradually increased up to 12 mA, at which point the abnormal movements disappeared. The TENS was then delivered daily in the following 11 months with 12 h of washout at night, and a gradual decrease in intensity to 6 mA, with continuous improvement (52). Another group reported two cases of confirmed functional propriospinal myoclonus treated with TENS. In the first participant, TENS was applied to the L2–4 dermatome with parameters of 71 mA intensity, 60 Hz frequency and 350 ms pulses, which led to improvements in walking and standing. The TENS treatment was discontinued due to allergic skin reaction and was replaced by direct stimulation of cutaneous femoris lateralis nerve, with a positive effect on standing ability. Since this is a pure sensory nerve, although stimulation intensity used could activate muscle, the sensory component was considered the main factor for the improvement with TENS. The second participant received TENS over the L3-S1 dermatome using stimulation parameters of 2 mA, 60 Hz, and 450 ms pulses. Myoclonus and gait unsteadiness were relieved only for a few days with the use of TENS, which was interrupted after the effects stopped (50).

There are only open-labeled studies evaluating the use of TENS in FMD. The co-existence of sensory and motor effects with this stimulation raises the possibility that the improvements could be due to placebo effects. However, using TENS alone or in combination with other tools can potentially be a promising method for treatment of FMD.

FES produces muscle contraction from peripheral nerve stimulation or motor point stimulation to provide functional movements. Typically, FES intervention is performed in combination with task specific functional movement where the voluntary effort of the subject is superimposed with electrical stimulation. It is a standard intervention for correction of drop foot for individuals with multiple sclerosis or stroke (53). A recent clinical guideline classifies the evidence level as “strong” for FES application in patients with stroke to improve gait speed, mobility, and dynamic balance (54). However, the parameters recommended in the guidelines were heterogenous and tailored to each patient. Hence, standardized studies are still needed to define an optimized protocol.

Modulation of the central nervous system is believed to play a role when applying FES for motor rehabilitation in conditions such as stroke and spinal cord injury (55, 56). Changes in diffusion tension imaging measured with MRI, functional MRI and EEG measurements have been associated with neurological improvement after FES, when used alone or in combination with task specific training in patients with stroke (57, 58). Although FES is more commonly used for motor rehabilitation following conditions such as stroke and spinal cord injury, other potential applications of this technique have been recently reported in movement disorders and functional neurological disorders (15, 59). Some of the results are summarized below.

One study reported a case of functional arm paresis treated with FES that comprised of a single 30-minute session of 30 Hz electrical stimulation applied to the right median, ulnar, and radial nerves, with a burst pattern (4 s on, 6 s off) to simulate voluntary muscle contractions and demonstrate limb movements to the patient. The minimum stimulus intensities used to induce visible muscle contractions were 10–18 mA. The patient had no immediate change in symptoms after treatment but reported gradual improvement over several weeks and full recovery by six months (59). Another report studied three patients with refractory fixed equinovarus dystonia. In one case, the patient was managed with a combination of botulinum toxin injections and FES of ankle muscles and showed long-lasting benefits for up to 12 months. The other two cases had more severe impairments, with one of them presenting with severe, fixed, soft tissue contractures. These two cases were not responsive to FES alone but responded to combination therapies including more invasive interventions like tibial nerve block, serial casting and joint manipulation under anesthesia (60). Another study discusses a case of functional paresis that received daily treatment with FES applied to the weak quadriceps and the paralyzed tibialis anterior muscles for two weeks. This intervention improved the function of the quadriceps and reversed the paralysis of the tibialis anterior muscle. The improvement in the functional abilities was documented through the use of quantitative measures of muscle strength as well as a computerized analysis of EMG signals (61).

There is limited evidence for the use of peripheral electrical stimulation in functional neurological disorders. In FMD, it could potentially be useful in patients with restriction of movements, most commonly fixed dystonia. However, specifics of the treatment, such as stimulation parameters, were not clearly described in the literature, which makes the interpretation of the results difficult. Large studies with better study designs, including standardized parameters and appropriate sham control are needed to evaluate the effectiveness of peripheral stimulation as a therapeutic modality for FMD.

## Final remarks and future directions

Since FMD is related to disturbances in functional brain connections rather than structural lesions, neuromodulation is potentially an effective treatment for FMD that remains underexplored. Most of the studies reported in the literature used TMS for neuromodulation with some promising results (29–31, 33). However, tDCS, TENS and FES have also shown some potential for treating FMD (44, 45, 51). Overall, the protocols of the studies reviewed (Table 1) were heterogeneous, and the underlying mechanisms were unclear. The motor or sensory effects of the stimuli may have produced a placebo effect. Most of the rTMS studies that involved a low number of stimuli or frequency (e.g., 0.25 Hz) below that typically used for neuromodulation (1 Hz) could be associated with placebo effect, whereas studies with stimulation of non-motor cortical areas and a higher frequency and longer stimulation duration could potentially be linked to neuromodulatory effects. Studies with larger sample sizes with proper sham controls are needed to prove the efficacy of central and peripheral neuromodulation techniques in FMD. Although designing an appropriate sham control is particularly challenging, since it must generate similar body sensations (on the scalp, skin, or muscles) without exerting a neurostimulation effect as the real intervention. Future studies using neurophysiological methods such as TMS, fMRI, or electroencephalography would also be important to elucidate the mechanisms of action of these neuromodulatory interventions.

**Table 1:** Summary of the neuromodulation studies in FMD.

Study	Technique	No. of patients and clinical presentation	Target	Protocol	Effects and outcome measures	Follow-up duration
Garcin (2013) Open-labelled	rTMS	24 patients dystonia, myoclonus, tremor, parkinsonism	Motor cortex	Single session of 20 stimuli of rTMS at 0.25 Hz, 120% RMT	Improvement in FMD score immediately after and in CGI at follow-up	19.8 months (median duration)
Garcin (2017) RCT, single blinded	rTMS	33 patients dystonia, myoclonus, tremor, parkinsonism, Stereotypes	Motor cortex and spinal nerve root	Single session of 20 stimuli of rTMS at 0.25 Hz and 120% RMT	Improvement in FMD score immediately after both real and sham conditions and CGI at follow-up	6 months – 1 year
Shah (2015) Open-labelled	rTMS	6 patients, dystonia, myoclonus, gait disorder	Motor cortex and premotor cortex	5 days of rTMS at 0.33 Hz, 90% RMT	Improvement in physical domain and reduction in psychological domain of WHOQOL-BREF	1 month
Taib (2019) RCT, double-blinded	rTMS	18 patients, functional tremor	Motor cortex	Phase I: 5 days of rTMS at 1 Hz, intensity of 90% RMT	Improvement in the PMDRS and CGI scorein the real compared to the sham group	2 months
Taib (2019) RCT, double-blinded	rTMS	17 patients, functional tremor	Motor cortex	Phase II: 3 weekly rTMS at 1 Hz, intensity of 90% RMT with 1 h hypnosis section	Improvement in the PMDRS and CGI score in the previous real compared to the previous sham group. No change HADS or HDRS scores in both groups.	12 months
Dafotakis (2011) Open-labelled	rTMS	8 patients, tremor	Motor cortex	Single session of 30 rTMS stimuli at 0.2 Hz. First half at 120% RMT, second half at 140% RMT	Reduction of tremor frequency—kinematic ultrasonic sensors recordings	3 moths – 1 year
Spagnolo (2021) Open-labeled	iTBS	6 patients, dystonia, myoclonus, gait disorder, tremor	Left prefrontal cortex	3 iTBS sessions of 600 pulses in 50 Hz bursts of 3 pulses, separated by 200 ms for 2 s followed by 8 s of no stimulation over 190 s	Improvement in the S-FMDRS, decreased front-amygdala connectivity and reactivity to emotional stimuli in fMRI	24 h
Demartini (2019) RCT, double-blinded	tDCS	9 patients dystonia, myoclonus, gait disorder and 7 healthy controls	Right posterior parietal cortex	single anodal tDCS session at 1.5 mA intensity for 20 min	Improvement in the heartbeat detection task (interoceptive sensitivity) after real and sham stimulation	none
Park (2021) Controlled trial	tDCS	5 patients, dystonia, myoclonus, gait disorder, tremor	Right temporoparietal junction	2 sessions of anodal tDCS at 2.0 mA intensity for 20 min + yoga practice	Improvement in CGI score after both real and sham stimulation. No difference in mRS and SSCI-8.	25 days
Ferrara (2011) Open-labelled	TENS	15 patients, dystonia, myoclonus, gait disorder, tremor	Muscles affected by FMD	TENS in 2 s trains, separated by 2 s pauses, at 150 Hz for 30 min daily	Improvement in PMDRS and in Patient-rated PMD severity	4 months

CGI, Clinical global impression; FMD, Functional movement disorder; FMD score, Abnormal Involuntary Movement Scale and Burke– Fahn–Marsden Scale; fMRI, functional magnetic resonance imaging; HADS, Hospital Anxiety and Depression; HDRS, Hamilton Depression Rating Scale; mRS, modified Rankin scale; iTBS, Intermittent theta burst stimulation; mRS, modified Rankin scale; PMD, Psychogenic movement disorder; PMDRS, Psychogenic Movement Disorders Rating Scale; RCT, Randomized controlled trial; RMT, Resting motor threshold; rTMS, Repetitive transcranial magnetic stimulation; S-FMDS, Simplified- Functional Movement Disorders Rating Scale; SSCI-8, Stigma Scale for Chronic Illness; tDCS, Transcranial direct current stimulation; TENS, transcutaneous electrical nerve stimulation;WHOQOL, The World Health Organization quality of life scale.

Also, the clinical presentation of FMD cases in the studies reviewed included a broad spectrum of movements, frequently with different types of movements in the same patient. The outcome measures used were not uniform, which makes it difficult to compare the results between studies and to standardize protocols for clinical use. In the future, more extensive studies comparing previously tested protocols with more uniform populations and longer follow-up times would help to clarify what type of neurostimulation is more effective for FMD. Moreover, tailoring different approaches for the diverse presentations of FMD is also a potential direction to investigate further. For example, peripheral electrical stimulation may be more appropriate for fixed dystonia, whereas rTMS might be better suited for treating functional tremor.

Lastly, there are newer modalities of neuromodulation that have shown promising results. For example, preliminary results using low-intensity focused ultrasound stimulation (TUS) has shown positive outcomes in increasing motor cortex excitability for up to 1 h (62). TUS has the advantage of being more focal than TMS and is able to modulate deeper brain structures compared to TMS or tDCS, which creates the possibility of stimulating deep brain areas including the thalamus and the basal ganglia (63). Clinical studies with TUS are still sparse, but there are protocols tested in patients with epilepsy or Alzheimer's disease (64). While there are TUS studies for movement disorders in animal models, there are no published studies of TUS in patients with movement disorders. It might be worth exploring the effects of TUS as a therapeutic modality in FMD.

## Conclusions

There might be a potential role of neuromodulation techniques as a therapeutic modality in the rehabilitation of patients with FMD, either by itself or in combination with other treatment modalities. Studies with larger sample size, better study design, appropriate sham control, more homogenous population and outcome measures, and longer follow-ups are needed to establish its efficacy for the treatment of FMD.

## References

[1] Garcin B. Motor functional neurological disorders: an update. Rev Neurol (Paris). (2018) 174(4):203–11. 10.1016/j.neurol.2017.11.003 29609961

[2] Lidstone SC Nassif W Juncos J Factor SA Lang AE. Diagnosing functional neurological disorder: seeing the whole picture. CNS Spectr. (2021) 26(6):593–600. 10.1017/S1092852920001996 33161935

[3] Hallett M Aybek S Dworetzky BA McWhirter L Staab JP Stone J. Functional neurological disorder: new subtypes and shared mechanisms. Lancet Neurol. (2022) 21(6):537–50. 10.1016/S1474-4422(21)00422-1 35430029 PMC9107510

[4] Lang AE. General overview of psychogenic movement disorders: epidemiology, diagnosis, and prognosis. In: Hallett M Fahn S Jankovic J Lang AE Cloninger CR Yudofsky SC, editors. Psychogenic movement disorders neurology and neuropsychiatry. Philadelphia: Lippincott Williams & Wilkins (2006). p. 35–41.

[5] Morgante F Edwards MJ Espay AJ. Psychogenic movement disorders. Continuum (Minneap Minn). (2013) 14(5):1383–96. 10.1212/01.CON.0000436160.41071.79 PMC4234133 24092294

[6] Carson A Lehn A. Epidemiology. Handb Clin Neurol. (2016) 139:47–60. 10.1016/B978-0-12-801772-2.00005-9 27719864

[7] Espay AJ Aybek S Carson A Edwards MJ Goldstein LH Hallett M Current concepts in diagnosis and treatment of functional neurological disorders. JAMA Neurol. (2018) 75(9):1132. 10.1001/jamaneurol.2018.1264 29868890 PMC7293766

[8] LaFaver K. Treatment of functional movement disorders. Neurol Clin. (2020) 38(2):469–80. 10.1016/j.ncl.2020.01.011 32279721

[9] Schwingenschuh P Espay A. Functional tremor. J Neurol Sci. (2022) 7:120208. 10.1016/j.jns.2022.120208 35306423

[10] Dallocchio C Tinazzi M Bombieri F Arnó N Erro R. Cognitive behavioural therapy and adjunctive physical activity for functional movement disorders (conversion disorder): a pilot, single-blinded, randomized study. Psychother Psychosom. (2016) 85(6):381–3. 10.1159/000446660 27744440

[11] Nielsen G Ricciardi L Demartini B Hunter R Joyce E Edwards MJ. Outcomes of a 5-day physiotherapy programme for functional (psychogenic) motor disorders. J Neurol. (2015) 262(3):674–81. 10.1007/s00415-014-7631-1 25557282

[12] Nielsen G Buszewicz M Stevenson F Hunter R Holt K Dudziec M Randomised feasibility study of physiotherapy for patients with functional motor symptoms. J Neurol Neurosurg Psychiatry. (2017) 88(6):484–90. 10.1136/jnnp-2016-314408 27694498

[13] Schmidt T Ebersbach G Oelsner H Sprock A König IR Bäumer T Evaluation of individualized multi-disciplinary inpatient treatment for functional movement disorders. Mov Disord Clin Pract. (2021) 8(6):911–8. 10.1002/mdc3.13268 34401405 PMC8354066

[14] Perez DL Edwards MJ Nielsen G Kozlowska K Hallett M LaFrance WC Jr. Decade of progress in motor functional neurological disorder: continuing the momentum. J Neurol Neurosurg Psychiatry. (2021) 92(6):668–77. 10.1136/jnnp-2020-323953 PMC8440656 33722822

[15] Taylor PN Sampson T Beare B Donavon-Hall M Thomas PW Marques E The effectiveness of peroneal nerve functional electrical simulation for the reduction of bradykinesia in Parkinson’s disease: a feasibility study for a randomised control trial. Clin Rehabil. (2021) 35(4):546–57. 10.1177/0269215520972519 33826449

[16] Shin HE Kim M Lee D Jang JY Soh Y Yun DH Therapeutic effects of functional electrical stimulation on physical performance and muscle strength in post-stroke older adults: a review. Ann Geriatr Med Res. (2022) 26(1):16–24. 10.4235/agmr.22.0006 35313099 PMC8984173

[17] McWhirter L Carson A Stone J. The body electric: a long view of electrical therapy for functional neurological disorders. Brain. (2015) 138(4):1113–20. 10.1093/brain/awv009 25711123

[18] Gelauff JM Dreissen YEM Tijssen MAJ Stone J. Treatment of functional motor disorders. Curr Treat Options Neurol. (2014) 16(4):286. 10.1007/s11940-014-0286-5 24566832

[19] Sasikumar S Strafella AP. The neuroimaging evidence of brain abnormalities in functional movement disorders. Brain. (2021) 144(8):2278–83. 10.1093/brain/awab131 33744915 PMC8418342

[20] Chen R Classen J Gerloff C Celnik P Wassermann EM Hallett M Depression of motor cortex excitability by low-frequency transcranial magnetic stimulation. Neurology. (1997) 48(5):1398–403. 10.1212/WNL.48.5.1398 9153480

[21] Pascual-Leone A Valls-Solé J Wassermann EM Hallett M. Responses to rapid-rate transcranial magnetic stimulation of the human motor cortex. Brain. (1994) 117(4):847–58. 10.1093/brain/117.4.847 7922470

[22] Lefaucheur JP Aleman A Baeken C Benninger DH Brunelin J Di Lazzaro V Evidence-based guidelines on the therapeutic use of repetitive transcranial magnetic stimulation (rTMS): an update (2014–2018). Clin Neurophysiol. (2020) 131(2):474–528. 10.1016/j.clinph.2019.11.002 31901449

[23] Fitzgerald PB. An update on the clinical use of repetitive transcranial magnetic stimulation in the treatment of depression. J Affect Disord. (2020) 276:90–103. 10.1016/j.jad.2020.06.067 32697721

[24] Blumberger DM Vila-Rodriguez F Thorpe KE Feffer K Noda Y Giacobbe P Effectiveness of theta burst versus high-frequency repetitive transcranial magnetic stimulation in patients with depression (THREE-D): a randomised non-inferiority trial. Lancet. (2018) 391(10131):1683–92. 10.1016/S0140-6736(18)30295-2 29726344

[25] Chen R Blumberger DM Fitzgerald PB, editors. A practical guide to TMS neurophysiology and treatment studies. New York, NY: Oxford University Press (2022).

[26] Berlow YA Zandvakili A Philip NS. Low frequency right-sided and high frequency left-sided repetitive transcranial magnetic stimulation for depression: the evidence of equivalence. Brain Stimulat. (2020) 13(6):1793–5. 10.1016/j.brs.2020.10.005 PMC7552991 33065359

[27] Latorre A Rocchi L Berardelli A Bhatia KP Rothwell JC. The use of transcranial magnetic stimulation as a treatment for movement disorders: a critical review. Mov Disord. (2019) 34(6):769–82. 10.1002/mds.27705 31034682

[28] Garcin B Roze E Mesrati F Cognat E Fournier E Vidailhet M Transcranial magnetic stimulation as an efficient treatment for psychogenic movement disorders. J Neurol Neurosurg Psychiatry. (2013) 84(9):1043–6. 10.1136/jnnp-2012-304062 23385844

[29] Garcin B Mesrati F Hubsch C Mauras T Iliescu I Naccache L Impact of transcranial magnetic stimulation on functional movement disorders: cortical modulation or a behavioral effect? Front Neurol. (2017) 8:338. 10.3389/fneur.2017.00338 28769869 PMC5515822

[30] Shah BB Chen R Zurowski M Kalia LV Gunraj C Lang AE. Repetitive transcranial magnetic stimulation plus standardized suggestion of benefit for functional movement disorders: an open label case series. Parkinsonism Relat Disord. (2015) 21(4):407–12. 10.1016/j.parkreldis.2015.01.013 25737204

[31] Taib S Ory-Magne F Brefel-Courbon C Moreau Y Thalamas C Arbus C Repetitive transcranial magnetic stimulation for functional tremor: a randomized, double-blind, controlled study. Mov Disord. (2019) 34(8):1210–9. 10.1002/mds.27727 31180620

[32] Dafotakis M Ameli M Vitinius F Weber R Albus C Fink G Der Einsatz der transkraniellen Magnetstimulation beim psychogenen Tremor - eine Pilotstudie. Fortschritte Neurol · Psychiatr. (2011) 79(04):226–33. 10.1055/s-0029-1246094 21480152

[33] Spagnolo PA Parker J Horovitz S Hallett M. Corticolimbic modulation via intermittent theta burst stimulation as a novel treatment for functional movement disorder: a proof-of-concept study. Brain Sci. (2021) 11(6):791. 10.3390/brainsci11060791 34203993 PMC8232716

[34] Kresojevic N Petrovic I Tomic A Svetel M Radovanovic S Kostic V. Transcranial magnetic stimulation in therapy of psychogenic neurological symptoms: two case reports. Mov Disord. (2010) 25(S2):S220. 10.1002/mds.23162

[35] Blades R Jordan S Becerra S Eusebio B Heatwole M Iovine J Treating dissociative post-traumatic stress disorder presenting as a functional movement disorder with transcranial magnetic stimulation targeting the cingulate gyrus. Neurol Sci. (2020) 41(8):2275–80. 10.1007/s10072-020-04433-2 32358703

[36] Naro A Pignolo L Billeri L Porcari B Portaro S Tonin P A case of psychogenic myoclonus responding to a novel transcranial magnetic stimulation approach: rationale, feasibility, and possible neurophysiological basis. Front Hum Neurosci. (2020) 14:292. 10.3389/fnhum.2020.00292 32848667 PMC7396578

[37] Bottemanne H Imadache K Pernet L de La Forest Divonne T English I Barron E Psychothérapie augmentée par rTMS pour les troubles neurologiques fonctionnels. L’Encéphale. (2022) 48(1):110–3. 10.1016/j.encep.2021.02.017 34099244

[38] Nitsche MA Cohen LG Wassermann EM Priori A Lang N Antal A Transcranial direct current stimulation: state of the art 2008. Brain Stimulat. (2008) 1(3):206–23. 10.1016/j.brs.2008.06.004 20633386

[39] Stagg CJ Antal A Nitsche MA. Physiology of transcranial direct current stimulation. J ECT. (2018) 34(3):144–52. 10.1097/YCT.0000000000000510 29877965

[40] Lefaucheur JP Antal A Ayache SS Benninger DH Brunelin J Cogiamanian F Evidence-based guidelines on the therapeutic use of transcranial direct current stimulation (tDCS). Clin Neurophysiol. (2017) 128(1):56–92. 10.1016/j.clinph.2016.10.087 27866120

[41] Koenigs M Grafman J. The functional neuroanatomy of depression: distinct roles for ventromedial and dorsolateral prefrontal cortex. Behav Brain Res. (2009) 201(2):239–43. 10.1016/j.bbr.2009.03.004 19428640 PMC2680780

[42] Dell’Osso B Dobrea C Arici C Benatti B Ferrucci R Vergari M Augmentative transcranial direct current stimulation (tDCS) in poor responder depressed patients: a follow-up study. CNS Spectr. (2014) 19(4):347–54. 10.1017/S1092852913000497 23962405

[43] Brunoni AR Valiengo L Baccaro A Zanão TA de Oliveira JF Goulart A The sertraline vs electrical current therapy for treating depression clinical study: results from a factorial, randomized, controlled trial. JAMA Psychiatry. (2013) 70(4):383. 10.1001/2013.jamapsychiatry.32 23389323

[44] Demartini B Volpe R Mattavelli G Goeta D D’Agostino A Gambini O. The neuromodulatory effect of tDCS in patients affected by functional motor symptoms: an exploratory study. Neurol Sci. (2019) 40(9):1821–7. 10.1007/s10072-019-03912-5 31044320

[45] Park JE Hong JY Lee SY. Transcranial direct current stimulation and yoga for functional movement disorders. Neurologist. (2021) 26(6):231–6. 10.1097/NRL.0000000000000345 34734899

[46] Mima T Oga T Rothwell J Satow T Yamamoto J-i Toma K Short-term high-frequency transcutaneous electrical nerve stimulation decreases human motor cortex excitability. Neurosci Lett. (2004) 355(1–2):85–8. 10.1016/j.neulet.2003.10.045 14729241

[47] Johnson MI Paley CA Jones G Mulvey MR Wittkopf PG. Efficacy and safety of transcutaneous electrical nerve stimulation (TENS) for acute and chronic pain in adults: a systematic review and meta-analysis of 381 studies (the meta-TENS study). BMJ Open. (2022) 12(2):e051073. 10.1136/bmjopen-2021-051073 35144946 PMC8845179

[48] Tinazzi M Farina S Bhatia K Fiaschi A Moretto G Bertolasi L TENS For the treatment of writer’s cramp dystonia: a randomized, placebo-controlled study. Neurology. (2005) 64(11):1946–8. 10.1212/01.WNL.0000163851.70927.7E 15955950

[49] Munhoz RP Hanajima R Ashby P Lang AE. Acute effect of transcutaneous electrical nerve stimulation on tremor. Mov Disord. (2003) 18(2):191–4. 10.1002/mds.10311 12539214

[50] Wojtecki L Groiss S Scherfeld D Albrecht P Pollok B Elben S Transient improvement of psychogenic (proprio-)spinal-like myoclonus to electrical nerve stimulation. Mov Disord. (2009) 24(13):2024–5. 10.1002/mds.22710 19645072

[51] Ferrara J Stamey W Strutt AM Adam OR Jankovic J. Transcutaneous electrical stimulation (TENS) for psychogenic movement disorders. J Neuropsychiatry Clin Neurosci. (2011) 23(2):141–8. 10.1176/jnp.23.2.jnp141 21677241

[52] Maltête D Verdure P Roze E Vidailhet M Apartis E Bellow F TENS For the treatment of propriospinal myoclonus. Mov Disord. (2008) 23(15):2256–7. 10.1002/mds.22315 18823015

[53] Taylor P Humphreys L Swain I. The long-term cost-effectiveness of the use of Functional Electrical Stimulation for the correction of dropped foot due to upper motor neuron lesion. J Rehabil Med. (2013) 45(2):154–60. 10.2340/16501977-1090 23303521

[54] Johnston TE Keller S Denzer-Weiler C Brown L. A clinical practice guideline for the use of ankle-foot orthoses and functional electrical stimulation post-stroke. J Neurol Phys Ther. (2021) 45(2):112–96. 10.1097/NPT.0000000000000347 33675603

[55] Furlan JC Pakosh M Craven BC Popovic MR. Insights on the potential mechanisms of action of functional electrical stimulation therapy in combination with task-specific training: a scoping review. Neuromodulation Technol Neural Interface. (2022). 10.1111/ner.13403. [Epud ahead of print] 34031937

[56] Andrews RK Schabrun SM Ridding MC Galea MP Hodges PW Chipchase LS. The effect of electrical stimulation on corticospinal excitability is dependent on application duration: a same subject pre-post test design. J NeuroEngineering Rehabil. (2013) 10:51. 10.1186/1743-0003-10-51. PMC3688368 23758902

[57] Boespflug EL Storrs JM Allendorfer JB Lamy M Eliassen JC Page S. Mean diffusivity as a potential diffusion tensor biomarker of motor rehabilitation after electrical stimulation incorporating task specific exercise in stroke: a pilot study. Brain Imaging Behav. (2014) 8(3):359–69. 10.1007/s11682-011-9144-1 22203524

[58] Wilkins KB Owen M Ingo C Carmona C Dewald JPA Yao J. Neural plasticity in moderate to severe chronic stroke following a device-assisted task-specific arm/hand intervention. Front Neurol. (2017) 8:284. 10.3389/fneur.2017.00284 28659863 PMC5469871

[59] Burke MJ Isayama R Jegatheeswaran G Gunraj C Feinstein A Lang AE Neurostimulation for functional neurological disorder: evaluating longitudinal neurophysiology. Mov Disord Clin Pract. (2018) 5(5):561–3. 10.1002/mdc3.12651 30515447 PMC6207130

[60] Nadler M Cary I Symeon C. Difficulties in management of functional movement disorders: three illustrative cases. Mov Disord Clin Pract. (2021) 8(6):932–9. 10.1002/mdc3.13264 34401406 PMC8354088

[61] Khalil TM Abdel-Moty E Asfour SS Fishbain DA Rosomoff RS Rosomoff HL. Functional electric stimulation in the reversal of conversion disorder paralysis. Arch Phys Med Rehabil. (1988) 69(7):545–7. 10.5555/uri:pii:0003999388900548 3260476

[62] Zeng K Darmani G Fomenko A Xia X Tran S Nankoo J Induction of human motor cortex plasticity by theta burst transcranial ultrasound stimulation. Ann Neurol. (2022) 91(2):238–52. 10.1002/ana.26294 34964172

[63] Darmani G Bergmann TO Butts Pauly K Caskey CF de Lecea L Fomenko A Non-invasive transcranial ultrasound stimulation for neuromodulation. Clin Neurophysiol. (2022) 135:51–73. 10.1016/j.clinph.2021.12.010 35033772

[64] Sarica C Nankoo JF Fomenko A Grippe TC Yamamoto K Samuel N Human studies of transcranial ultrasound neuromodulation: a systematic review of effectiveness and safety. Brain Stimulat. (2022) 15(3):737–46. 10.1016/j.brs.2022.05.002 35533835

